# Supplementary material for: Phase 2 Trial (POLA Study) of Lurbinectedin plus Olaparib in Patients with Advanced Solid Tumors: Results of Efficacy, Tolerability, and the Translational Study
Source: Cancers (Basel). 2022 Feb 12;14(4):915. doi: 10.3390/cancers14040915 (PMC8870416; doi:10.3390/cancers14040915)

Manuscript Supplementary Material

Title:

Phase II trial (POLA study) to evaluate the efficacy and tolerability of lurbinectedin plus olaparib in patients with advanced solid tumors: results from the translational study

Authors:

Andres Poveda, Raquel Lopez-Reig, Ana Oaknin, Andres Redondo, Maria Jesus Rubio, Eva Guerra, Lorena Fariñas-Madrid, Alejandro Gallego, Victor Rodriguez-Freixinos, Antonio Fernandez-Serra, Oscar Juan, Ignacio Romero, Jose A Lopez-Guerrero

Corresponding author:

Andres Poveda, MD

[1] Oncogynecologic Department, Initia Oncology, Hospital Quironsalud Avda Blasco Ibañez, 14, 46 010-Valencia, Spain. E-mail: apoveda@initiaoncologia.com

Table of Contents

Supplementary Materials and Methods 3
Mutation-based classification: HRD status 3
CN patterns comparison between cancer type 3
Assessing CN amplification and losses at gene level. 3
Table S1. Best overall response rate by tumor type 3
Figure S1. Consort diagram. 5
Figure S2. 6
A. Kaplan-Meier curves for the whole population 6
B. Kaplan-Meier curves of progression-free survival according to histology (ovarian vs. endometrium) 7
C. Swimmer plot of progression-free survival by tumor type 7
Figure S3. 9
A. Kaplan-Meier curves of Overall survival for the whole population. 9
B. Kaplan-Meier curves of Overall survival according to histology (ovarian vs. endometrium) 10
Figure S4: Comparison of GI patterns between cancer types. A) Total number of events(d=0.67), B) Total number of gains(d=0.76), and C) Total number of losses (d=0.54),. Non-parametric Wilcoxon Signed Ranks Test was used. 11
Figure S5: Clinical implication of GI parameters regarding ORR in A) Global population (d=0.84), B) EC population (d=0.34), Non-parametric Wilcoxon Signed Ranks Test was used. 11
Figure S6: Clinical implication of GI parameters in OC population regarding CBR. A) Total number of events (d=0.62), and B) total number of gains (d=0.69). Non-parametric Wilcoxon Signed Ranks Test was used. 12

Supplementary Materials and Methods

Mutation-based classification: HRD status

For gene-based HRD status classification, genes that were considered to assign a patient as HRD were: BRCA1, BRCA2, BARD1, BRIP1, CHEK1, CHEK2, FAM175A, NBN, PALB2, ATM, MRE11A, RAD51B, RAD51C, RAD51D, RAD54L, SLX4, WRN, ATR.

CN patterns comparison between cancer type

When comparing EC and OC cohorts, differences at global GI (p=0.032) as well as at gains (p=0.0046) and losses (p=0.022) appeared. However, LOH events neither percentage of altered genome showed significant statistical differences. In all cases, OC population presented higher number of GI, both globally and parameter-dependent (S5).

Assessing CN amplification and losses at gene level.

In addition to the GI profiling, a custom panel was also designed to interrogate CN at gene level. Since *PTEN* loss and *EMSY* and *CCNE1* amplification have been described in these tumor types, the panel was reinforced in these regions with more probes. Hence, CN data analyzed by panel mops package were used. A total of 6 and 1 amplification were detected in CCNE1 and EMSY, respectively, while 5 patients presented *PTEN* loss among the EC population. Concerning CCNE1 amplifications, 4 were found in EC and 2 in OC, any of them coinciding with HRR mutation, being mutually exclusive. *PTEN* was also found in the two subpopulations, 2 events in EC and 3 in OC. These alterations were validated by MLPA, the gold standard technique, to assess CN at gene level. While amplification was confirmed in all cases, the validation of PTEN loss was not possible. Cases harboring these alterations have not shown correlation with response to PARPi. Additionally, a subanalysis stratifying OC patients according to the presence of CCNE1 amplification, HRR mutation or none of them was performed, also lacking significant results.

Table S1. Best overall response rate by tumor type

	Tumor type			
	Ovary[1] (N = 46 patients)		Endometrium (N = 26 patients)	
	N	%	N	%
**Complete Response**	0	0	1	3.8
**Partial Response**	3	6.5	3	11.5
**Stable Disease**	31	67.4	14	53.8
**Progressive Disease**	8	17.4	7	26.9
**NO Evaluable**	4	8.7	1	3.8

[1]Ovarian, fallopian tube and primary peritoneum tumors

Best Overall Response defined as the best response presented according to RECIST 1.1 criteria during the treatment period

Table S2. Long-term responders

Patient ID	PFS Evaluation (Censored YES/NO)	Response Assessment<br>PFS Event	PFS time	Long-Time Responder
01001	No	Progression	1.3479452	No
01002	No	Progression	2.5972603	No
01003	No	Progression	1.2164384	No
01004	No	Progression	4.6027397	No
01005	No	Progression	4.3726027	No
01006	No	Progression	13.347945	Yes
01008	No	Progression	1.3808219	No
02001	No	Progression	5.5890411	No
02003	No	Progression	2.1369863	No
02004	No	Progression	5.1287671	No
02005	Yes	.	0.0328767	No
02006	Yes	.	1.6767123	No
02007	No	Progression	9.9287671	Yes
02008	No	Progression	7.5287671	No
02009	No	Progression	5.9506849	No
02010	No	Progression	9.5342466	Yes
02011	Yes	.	23.243836	Yes
02012	No	Progression	19.364384	Yes
02013	No	Progression	4.8	No
02014	No	Progression	4.5369863	No
02015	No	Progression	2.7616438	No
02016	No	Progression	12.09863	Yes
02017	No	Progression	2.5643836	No
02018	No	Progression	5.5561644	No
02019	No	Death	17.687671	Yes
02020	No	Progression	5.030137	No
02021	No	Progression	1.7753425	No
02023	No	Progression	6.1808219	No
02024	No	Progression	1.6109589	No
02025	Yes	.	0.0328767	No
02026	No	Progression	6.1150685	No
03001	No	Progression	1.5123288	No
03002	No	Progression	6.8712329	No
03004	No	Progression	3.6821918	No
03005	Yes	.	1.8410959	No
03006	No	Progression	1.8410959	No
03007	Yes	.	8.6136986	No
03008	No	Progression	1.6767123	No
03009	No	Progression	1.9068493	No
03010	No	Progression	1.5780822	No
03011	No	Progression	3.0246575	No
03013	No	Progression	1.8410959	No
03014	Yes	.	15.978082	Yes
03015	No	Progression	9.3041096	Yes
03016	No	Progression	2.8931507	No
03017	No	Progression	4.6027397	No
03018	No	Progression	4.569863	No
03019	No	Progression	4.1753425	No
04001	No	Progression	3.0575342	No
04003	No	Progression	3.4191781	No
04004	No	Progression	2.9260274	No
04005	No	Progression	2.9260274	No
04006	No	Progression	1.4465753	No
04008	No	Progression	6.9369863	No
04009	No	Progression	9.7315068	Yes
04010	Yes	.	0.0328767	No
04011	No	Progression	1.3150685	No
04013	No	Progression	6.8054795	No
04014	Yes	.	0.0328767	No
04015	No	Progression	1.4465753	No
05003	No	Progression	5.2931507	No
05005	No	Progression	2.9917808	No
05006	No	Progression	12.953425	Yes
05007	No	Progression	4.2082192	No
05008	No	Progression	5.7534247	No
05009	No	Death	1.7424658	No
05010	No	Progression	4.6684932	No
05011	No	Progression	1.5780822	No
05012	No	Progression	5.9835616	No
05013	No	Progression	3.2219178	No

Patient ID	PFS Evaluation (Censored YES/NO)	Response Assessment<br>PFS Event	PFS time	Long-Time Responder
05014	No	Progression	2.9589041	No
05015	No	Progression	5.5561644	No
05016	Yes	.	9.1726027	Yes

Long term responders defined as those patients whose PFS is greater-equal the double estimated median PFS :4.54

Figure S1. Consort diagram.

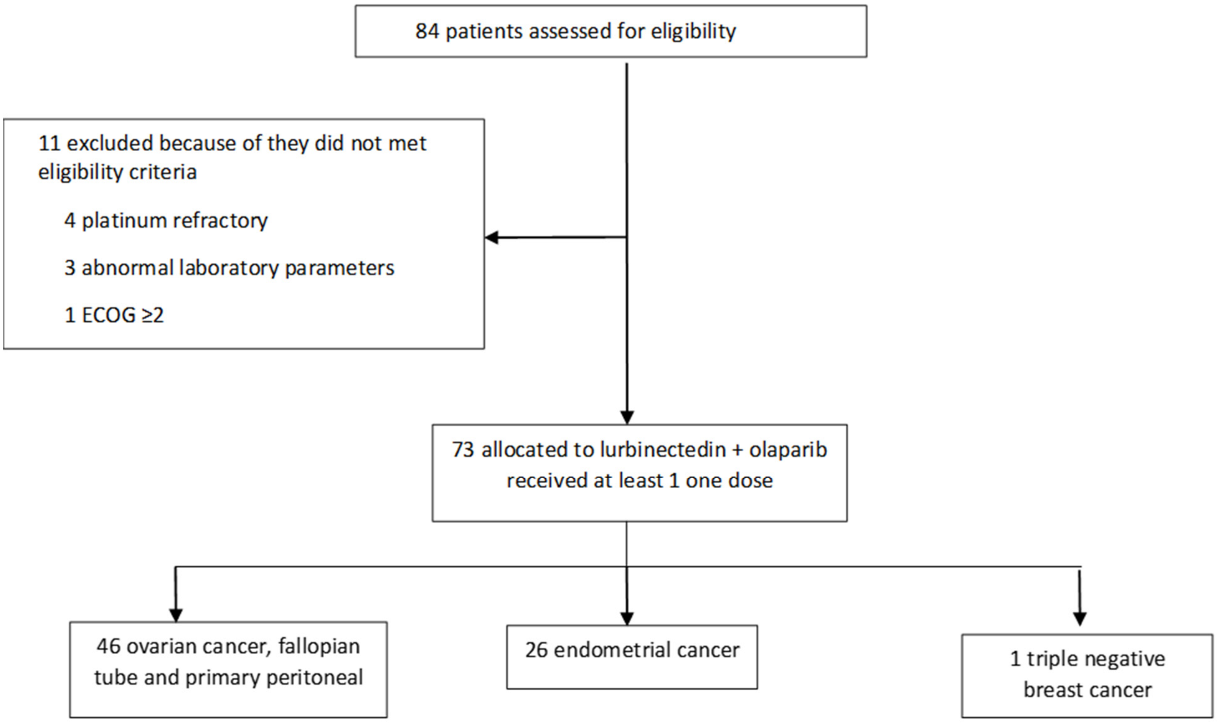

Figure S2.

A. Kaplan-Meier curves for the whole population

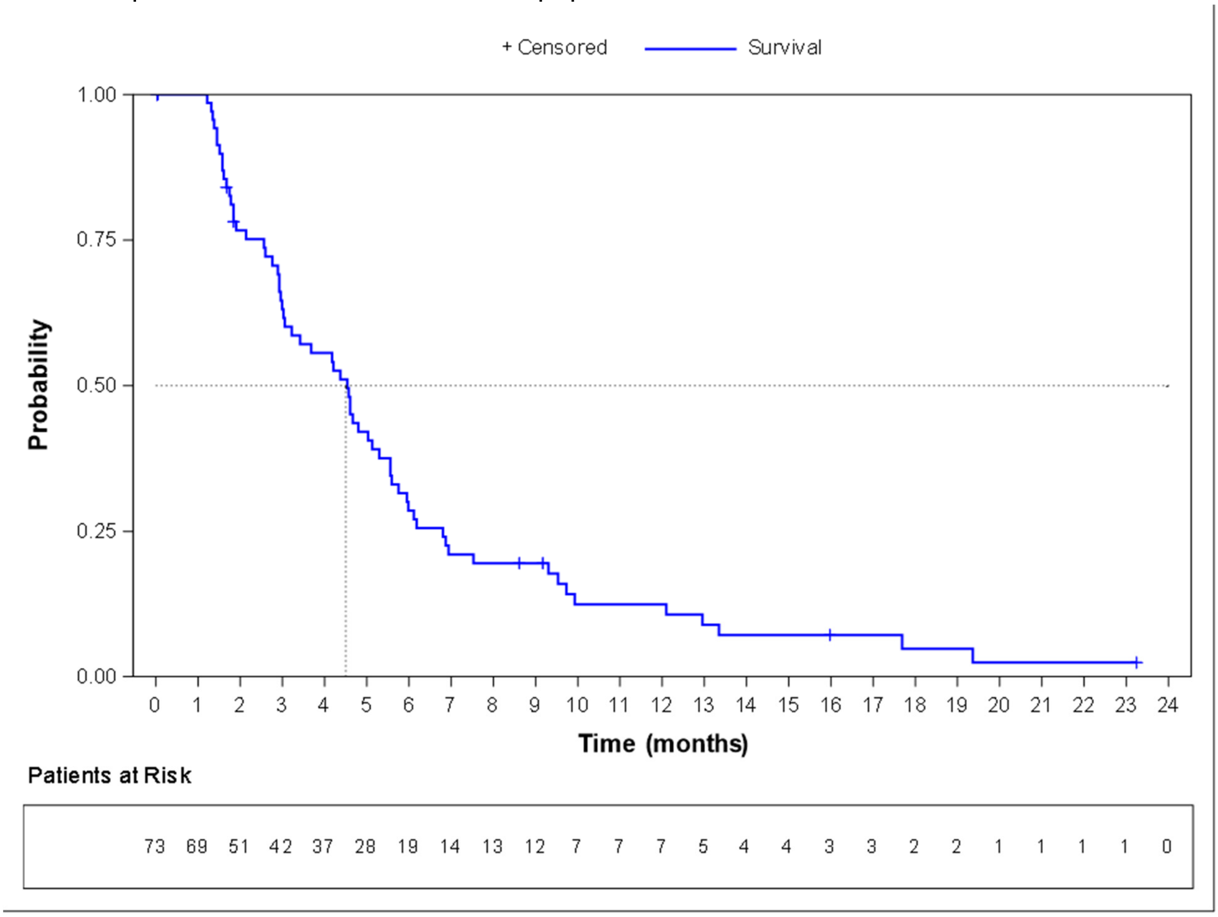

B. Kaplan-Meier curves of progression-free survival according to histology (ovarian vs. endometrium)

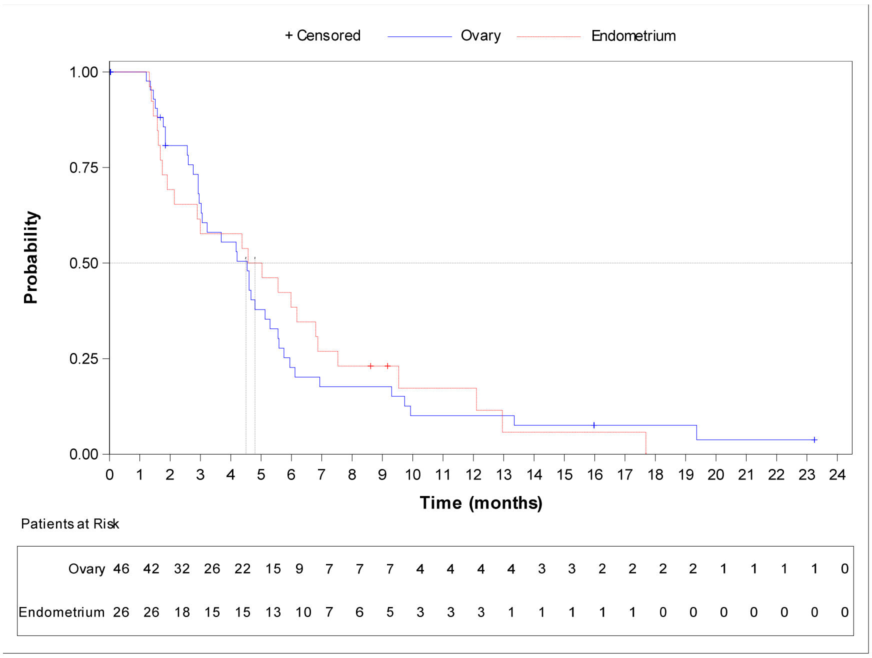

Ovary median PFS 4.5 months (95% CI, 3.0-5.1)
Endometrium median PFS 4.8 months (95% CI, 1.9-6.8)
HR 0.97 (CI 95%, 0.56-1.59); p = 0.852

C. Swimmer plot of progression-free survival by tumor type

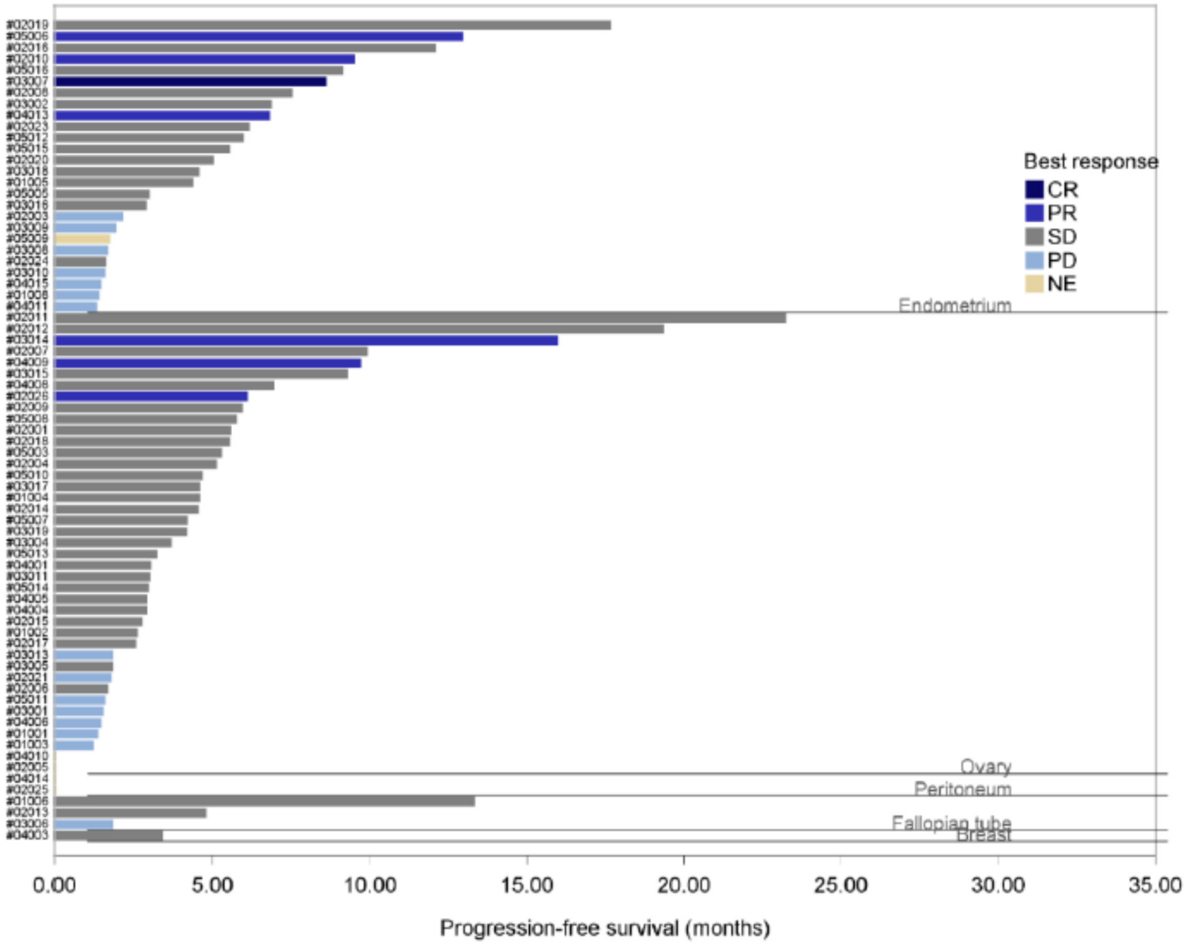

Patients who did not registered RECIST assessment during the study, their PFS was computed as censored at the time of inclusion plus one day.

Figure S3.

A. Kaplan-Meier curves of Overall survival for the whole population.

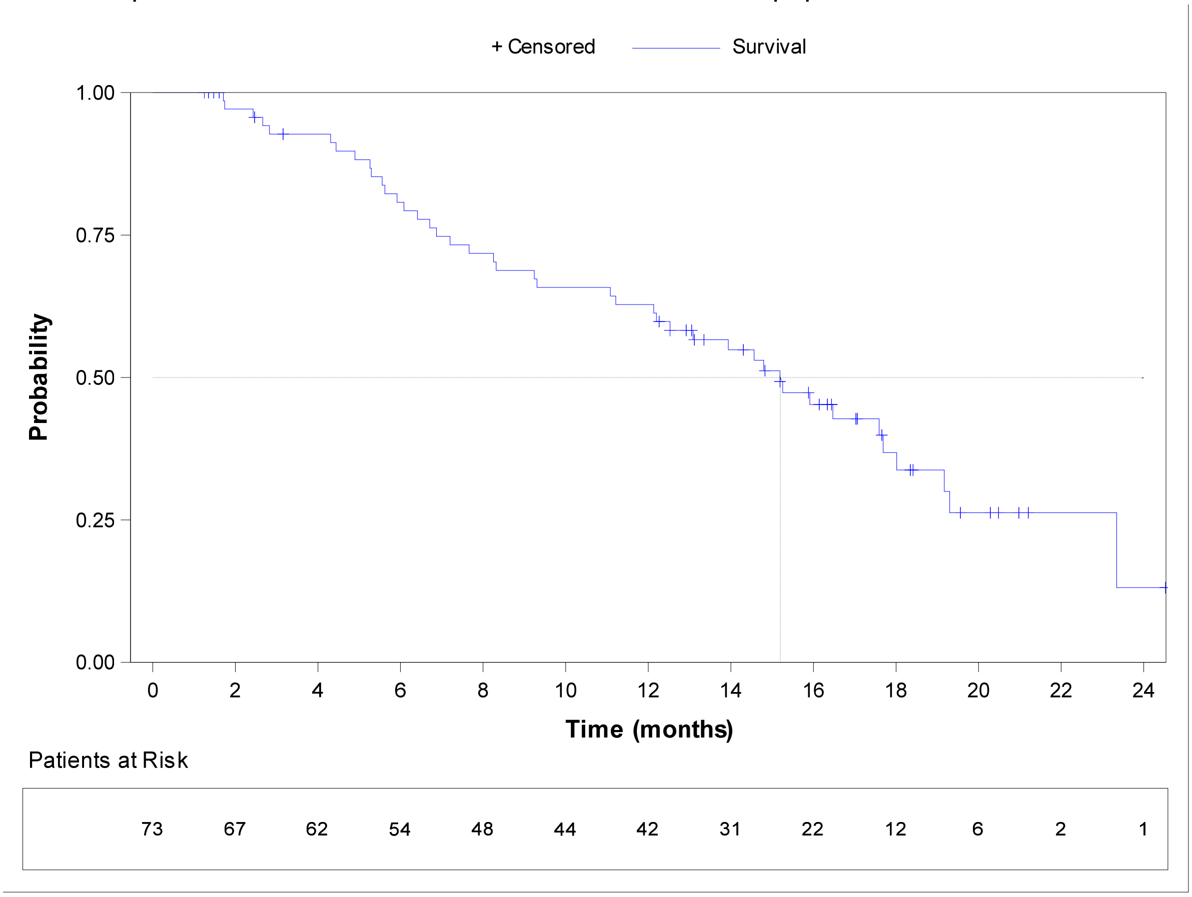

Median OS (95% CI) 15.19 months (12.13, 17.69)

B. Kaplan-Meier curves of Overall survival according to histology (ovarian vs. endometrium)

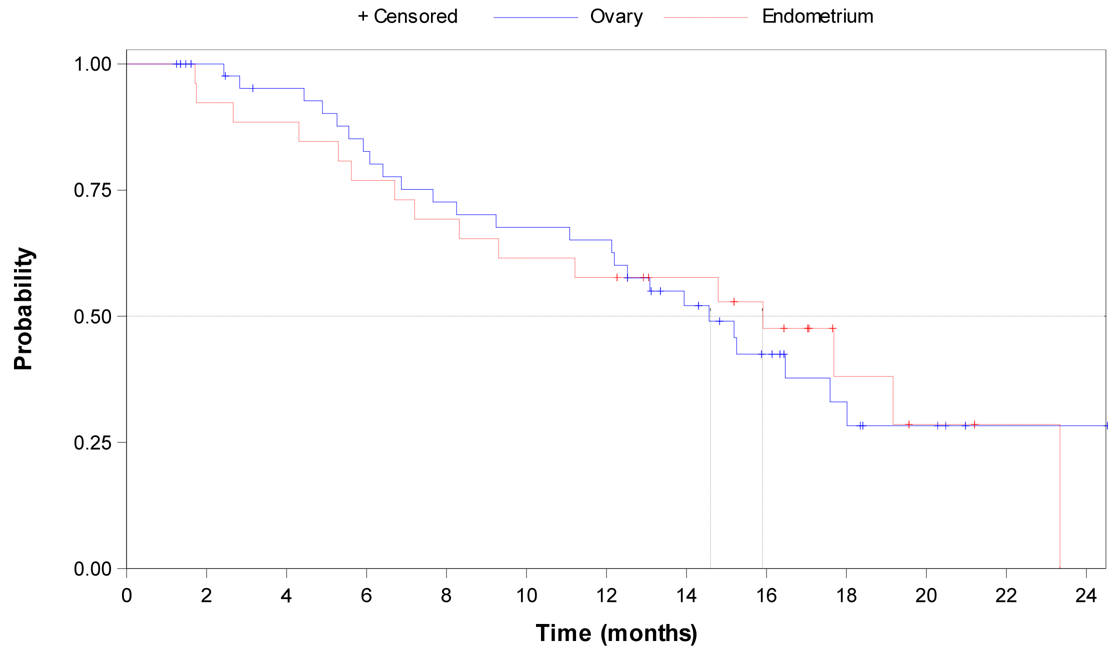

Patients at Risk

Ovary	46	42	38	33	29	27	26	18	12	7	4	1	1
Endometrium	26	24	23	20	18	16	15	12	9	4	2	1	0

Strata	Subjects	Event	% Events	Censored	% Censored	Median	CI 95% LL	CI 95% UL
Endometrium	26	16	61.5	10	38.5	15.9	7.2	23.3
Ovary	46	25	54.3	21	45.7	14.6	11.1	17.6

Test of Equality over Strata

Test	Chi-Square	DF	Pr > Chi-Square
Log-Rank	0.0015	1	0.9688

Cox regression results

Contrast	HR pvalue	HR Estimate	CI 95% LL	CI 95% UL
Endometrium vs 2	0.9688	0.988	0.526	1.852

Figure S4: Comparison of GI patterns between cancer types. A) Total number of events(d=0.67), B) Total number of gains(d=0.76), and C) Total number of losses (d=0.54),. Non-parametric Wilcoxon Signed Ranks Test was used.

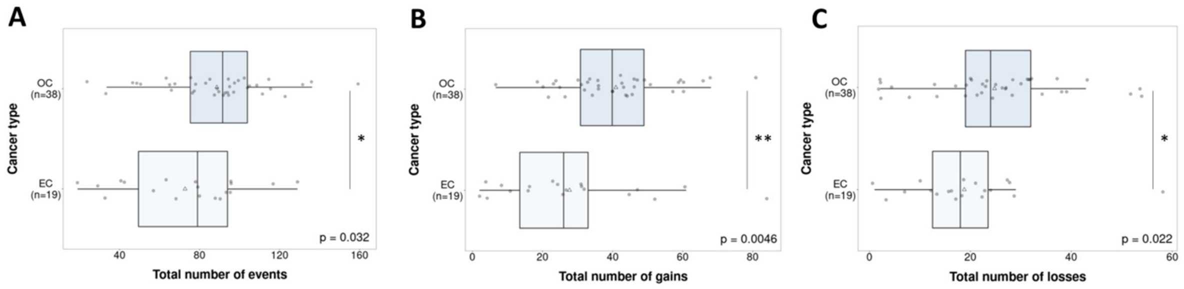

Figure S5: Clinical implication of GI parameters regarding ORR in A) Global population (d=0.84), B) EC population (d=0.34), Non-parametric Wilcoxon Signed Ranks Test was used.

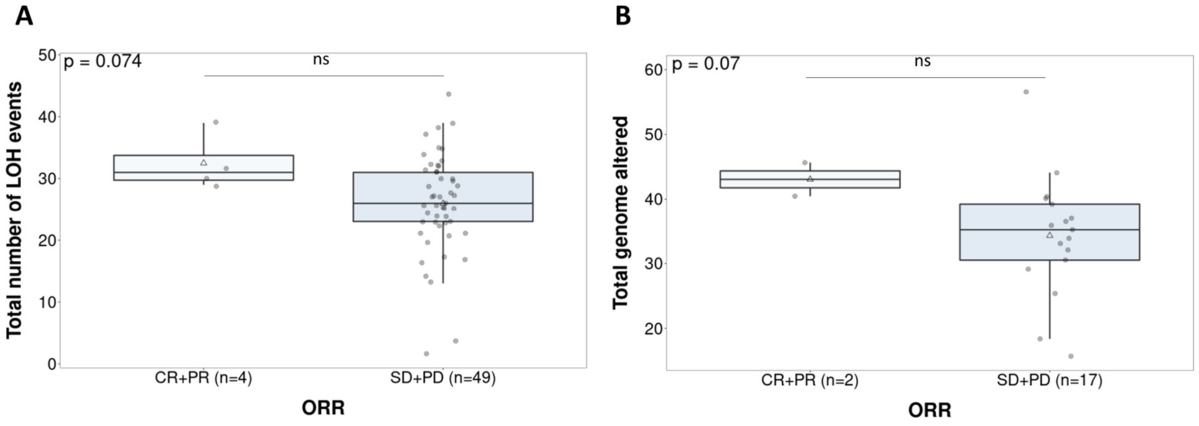

Figure S6: Clinical implication of GI parameters in OC population regarding CBR. A) Total number of events (d=0.62), and B) total number of gains (d=0.69). Non-parametric Wilcoxon Signed Ranks Test was used.

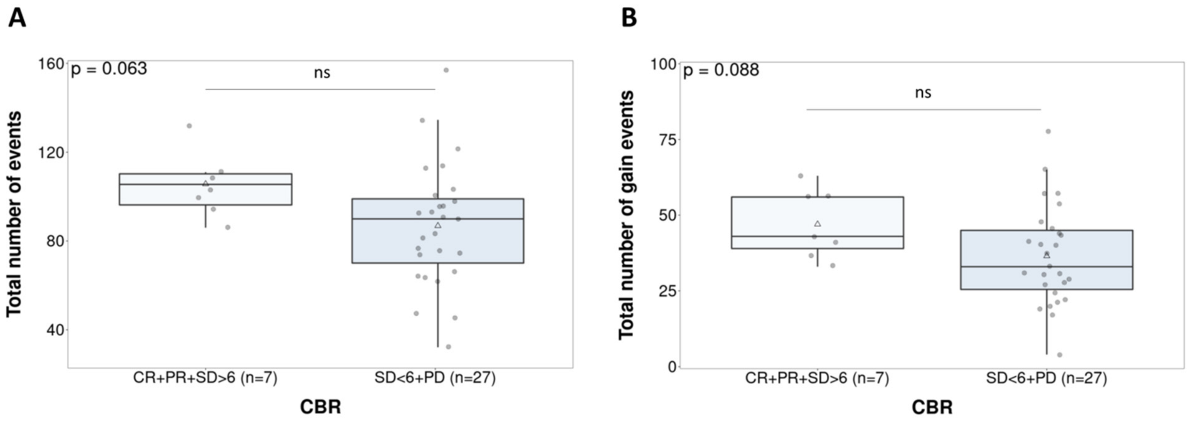

Supplement: Supplementary file 1 [file cancers-14-00915-s001.zip › cancers-1542473-supplementary.pdf]
